# Biodegradable Polymer Composites Based on Poly(butylene succinate) Copolyesters and Wood Flour

**DOI:** 10.3390/polym17070883

**Published:** 2025-03-26

**Authors:** Agnieszka Kozłowska, Krzysztof Gorący, Miroslawa El Fray

**Affiliations:** Department of Polymer and Biomaterials Science, Faculty of Chemical Technology and Engineering, West Pomeranian University of Technology, Al. Piastow 45, 71-311 Szczecin, Poland; agnieszka.kozlowska@zut.edu.pl (A.K.); kgoracy@zut.edu.pl (K.G.)

**Keywords:** biodegradable composites, poly(butylene succinate), wood–plastic composites, wood flour, dimerized fatty acid, dilinoleic acid, biodegradation

## Abstract

This study investigates the biodegradation behavior of poly(butylene succinate) (PBS) copolyesters containing dilinoleic acid (DLA) co-monomeric units and wood flour (WF) as a filler. PBS-DLA is a segmented thermoplastic elastomer (TPE), where the soft amorphous phase is formed by DLA ester segments, while the hard phase consists of crystallizable PBS domains. Wood–plastic composites (WPCs) were prepared with WF at weight fractions of 10%, 20%, 30%, and 40% wt. and analyzed in terms of surface morphology, chemical structure, mechanical performance, and thermal stability before and after biodegradation in soil conditions. The results of microscopic analysis confirmed that the PBS-DLA copolymer and its composites undergo surface biodegradation as manifested by increased surface roughness and microcrack formation, particularly in composites with a higher WF content. ATR FT-IR spectroscopy indicated oxidation and hydrolysis, supporting the hypothesis of progressive surface erosion. Mechanical tests showed a decline in tensile strength and elongation at break, with the most pronounced changes in composites containing 20% WF. Thermal analysis (DSC, DMTA, and TGA) confirmed that the PBS-DLA copolymer retains its thermoplastic elastomeric behavior after a 3-month biodegradation experiment. The storage modulus (E′) remained stable, while only minor variations in melting and crystallization temperatures were observed. These findings reinforce the hypothesis of surface erosion rather than a bulk degradation mechanism. Given their biodegradability and retained thermoplastic behavior, WPC composites based on PBS-DLA copolyester could be promising for eco-friendly applications where controlled degradation is desirable, such as in packaging, agriculture, or biodegradable consumer goods.

## 1. Introduction

Environmental pollution caused by plastic waste is one of the greatest challenges facing contemporary waste management. Conventional polymers, despite their wide range of applications and excellent user properties, are characterized by long degradation times, leading to their accumulation in terrestrial and aquatic ecosystems. Recent studies highlight the urgent need for sustainable alternatives to conventional plastics, emphasizing biodegradable polymers as a promising solution to this global problem [1,2,3]. It is estimated that since the 1950s, over 8 billion tons of plastic have been produced globally, the vast majority of which has not been recycled but has instead ended up in landfills or in the natural environment [1]. Particularly problematic are the waste materials from single-use packaging, which make up the dominant fraction of plastic municipal waste and are difficult to process [2]. As a result, these wastes can persist in the environment for hundreds of years, causing serious ecological consequences, including the degradation of marine ecosystems and the release of microplastics, which infiltrate the food chain [4].

In response to the issue of excessive consumption of conventional plastics, circular economy strategies are being implemented, aiming to reduce waste and increase recycling efficiency [2]. One of the key elements of these efforts is the search for alternative materials with a reduced environmental impact, including biodegradable plastics [2,3,4,5]. Biodegradable polymers can break down under the influence of microorganisms, leading to the formation of harmless end products such as carbon dioxide, water, and biomass [4,6]. The polymer degradation process occurs in three stages: initiation (oxidation and breaking of bonds in the macromolecule structure), fragmentation (formation of short polymer chains), and mineralization, in which low-molecular-weight products further break down into simple compounds [7]. Recent studies have identified esterases and lipases as key enzymes responsible for PBS degradation under mesophilic conditions, facilitating the hydrolytic cleavage of ester bonds and accelerating polymer breakdown [8].

Depending on their origin, biodegradable plastics are divided into natural polymers, such as starch, cellulose, and chitosan, and synthetic ones, including aliphatic polyesters [4,5]. The potential of natural polymers in sustainable applications has been widely studied, particularly in the context of their role in environmental remediation and their synergy with synthetic biodegradable materials [9]. Due to the presence of ester bonds in their structure, these polyesters exhibit a high susceptibility to enzymatic degradation and are considered one of the most promising groups of biodegradable materials [7,8,9,10,11,12,13]. The industrial applications of biodegradable polymers primarily include the packaging sector, agricultural applications, and medicine, including the manufacture of surgical threads and drug carriers [10]. At the same time, methods for their chemical synthesis are being developed, allowing for the modification of their properties and adaptation to specific applications [11]. The PBS processability is also a crucial factor influencing its industrial applications. Recent studies have provided a comprehensive overview of the thermal stability, melt processing conditions, and processability of PBS, highlighting its potential for large-scale applications [12]. In the context of organic recycling, it is particularly important that biodegradable polymers can undergo effective degradation under composting conditions, making them a viable alternative to conventional plastics [13].

Poly(butylene succinate) (PBS) is a biodegradable aliphatic polyester that is gaining increasing attention as a sustainable alternative to conventional synthetic polymers [14]. PBS is characterized by favorable mechanical properties, high processability, and good thermal resistance, making it an attractive material for industrial applications such as film production, fibers, and injection-molded components [12,14]. This polymer can be synthesized both petrochemically and from renewable resources, which increases its attractiveness in the context of a circular economy [15].

The biodegradability of PBS results from the presence of ester bonds in its structure, which are susceptible to enzymatic and microbiological hydrolysis. This material decomposes in various environments, including soil, water, and composting conditions, making it competitive with other biodegradable polymers, such as polylactic acid (PLA) or polyhydroxyalkanoates (PHA). Recent research has explored the role of microbial consortia in the biodegradation of PBS, demonstrating that targeted microbial communities can significantly enhance its degradation efficiency under controlled conditions [16]. Despite these advantages, PBS has also certain limitations, including low mechanical resistance in some applications and relatively high production costs, which stimulate research into its copolymerization and modifications, such as reinforcement with natural fillers [17].

In addition to traditional industrial applications, PBS is also used in biomedicine, where it is employed in the production of biodegradable implants, surgical threads, and controlled drug release systems. Its ability to biodegrade in the body makes it a promising material for modern medical applications [18,19,20].

PBS can be modified through copolymerization with various co-monomers, allowing for the adjustment of its mechanical and thermal properties along with its biodegradation. An example is poly(butylene succinate-co-butylene adipate) (PBSA), which exhibits greater flexibility and faster biodegradation compared to neat PBS, making it attractive for use in biodegradable packaging [21]. Another material is poly(butylene adipate-co-terephthalate) (PBAT), which, due to the presence of terephthalate units, shows high flexibility and good mechanical strength, making it widely used in biodegradable films [22]. Copolymerization of PBS with furanoate improves thermal stability and increases hydrophobicity, making the polymer resistant to degradation in moist environments [23]. Another method involves copolymerization with thiophenedicarboxylate, resulting in polymers with a dual crystalline structure, which enhances their mechanical and thermal properties [24]. Additionally, crosslinking PBS with fumaric acid leads to the formation of crosslinked structures with greater thermal stability and resistance to degradation [25].

The modification of PBS with a vegetable oil (VO) monomer, such as dilinoleic acid (DLA), often named as dimerized fatty acid, can lead to the formation of elastomeric segmented copolymers of the PBS-DLA type, where PBS serves as the hard segment and DLA as the soft segment. These types of copolymers exhibit favorable mechanical and thermal properties, combining the strength typical of engineering materials with the flexibility of rubber. By altering the ratio of hard and soft segments, their properties such as flexibility, hardness, and biodegradation rate can be controlled, making them a promising option for biodegradable applications [26]. Our earlier studies have shown that PBS-DLS polymers synthesized using an enzymatic catalyst (lipase B from *Candida antarctica*) have a more blocky segmental distribution and higher crystallinity in comparison with materials synthesized using a heterogeneous metalloorganic catalyst, which affected the differences in the length of hard segments and the crystallinity of such polymers [27]. Moreover, calculations of the degree of randomness (R) showed that PBS-DLS copolymers synthesized with metalloorganic catalyst have a random distribution (R close to 1) of the hard and soft segments within the copolymer structure.

Wood–plastic composites (WPCs) are becoming increasingly popular as ecological materials, combining the advantages of biodegradable polymers and renewable lignocellulosic resources. Wood flour, one of the most commonly used fillers, contributes to improving properties such as the stiffness, hydrophilicity, and biodegradability of the composite. Recent studies highlight the role of wood-derived fillers in improving the biodegradability and mechanical properties of polymer composites, particularly in PBS-based systems [28]. The literature indicates that a higher lignocellulosic filler content accelerates polymer degradation, although these effects depend on the distribution of the filler in the polymer matrix and its interaction with the environment [29,30].

Studies on WPC biodegradation suggest that lignocellulosic fillers can facilitate microbial colonization, accelerating degradation under natural conditions. Changes in the mechanical properties, roughness, and moisture content of the composite are also observed, which may affect the durability and functionality of these materials [29,31]. Therefore, it is essential to conduct a comprehensive study of the impact of lignocellulosic fillers on the biodegradation of PBS and to understand the degradation mechanisms under real environmental conditions.

In this context, studies were also conducted on the biodegradation and mechanical properties of agro-flour-filled polybutylene succinate (PBS) biocomposites, which demonstrated that the addition of agro-flour improved the biodegradability of the material while maintaining its mechanical strength. These findings highlight the potential of agro-flour-filled PBS as a promising biodegradable material [32].

The aim of this study was to determine the effect of wood flour addition on the biodegradation and physicochemical properties of PBS-DLA copolyester with a 50-50 wt.% ratio of hard to soft blocks [26]. WPC composite samples with varying filler content were subjected to three-month biodegradation tests under soil conditions, analyzing the mass loss, changes in surface morphology using stereoscopic and laser microscopy, contact angles, and FTIR spectral changes. Furthermore, the effect of biodegradation on the mechanical properties of the materials was evaluated. The obtained results will provide a better understanding of the biodegradation mechanisms of PBS-DLA copolymers and their composites, as well as assessing their suitability as ecological materials with a controlled service life.

## 2. Materials and Methods

### 2.1. Materials

The following reagents were used for the synthesis of the copolyester: succinic acid (SA) (Sigma-Aldrich, Germany), dilinoleic acid (DLA)—Pripol 1009 (Cargill Bioindustrial, Gouda, The Netherlands), 1,4-butanediol (BDO) (Sigma-Aldrich, Poznań, Poland), and the heterogenous titanium dioxide/silicon dioxide (TiO_2_/SiO_2_) catalyst C-94 (Huntsman, Deggendorf, Germany).

The wood flour (WF) JELUXYL WEHO 500 used as a filler was supplied by Jelu-Werk (Rosenberg, Germany) and derived from softwood species (spruce, fir). It was characterized by a bulk density of 140 kg/m^3^, a decomposition temperature above 170 °C, a pH of approximately 5.5, and a light yellow color. The material was in solid form and was sieved to achieve a particle size below 500 µm, ensuring uniform dispersion in the polymer matrix.

### 2.2. Synthesis of Copolymer and Composite Preparation

**Figure 1:**
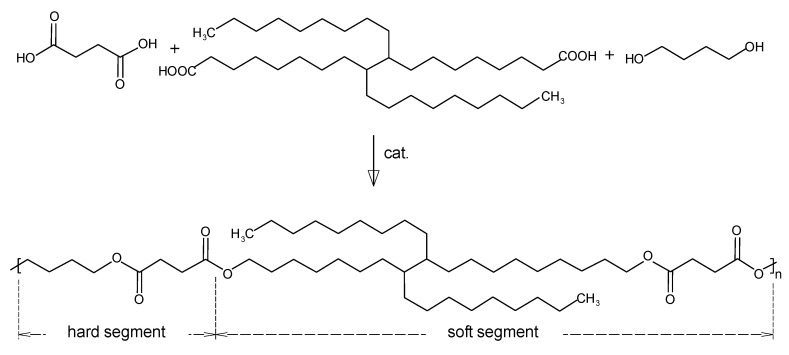
Schematic of the multiblock copolyester PBS-DLA synthesis.

PBS-DLA is a segmented copolyester composed of hard PBS segments and soft DLA segments (Figure 1). The copolymer composition was designed to achieve a 50-50 weight ratio of these segments, balancing mechanical performance and biodegradability [19].

The PBS-DLA (50-50 wt.%) copolyester was synthesized via a two-step melt polycondensation process in a stainless steel reactor equipped with a mechanical stirrer, nitrogen inlet, and vacuum system. In the first step (esterification), the monomers and catalyst were reacted under atmospheric pressure at 100–200 °C, with the continuous removal of water as the by-product. In the second step (polycondensation), the temperature was increased to 240–250 °C, and the pressure was gradually reduced to 0.4 hPa to remove volatile by-products and facilitate polymer chain growth. The reaction was carried out until a high-viscosity polymer melt was obtained. The final copolyester was extruded as a filament and granulated for further processing.

The polymer composites were prepared by incorporating wood flour (WF) into the synthesized PBS-DLA copolyester. The WF was pre-dried at 105 °C for 24 h and sieved to a particle size below 500 µm. The components were premixed and processed in a Thermo Fisher Scientific (Waltham, MA, USA) co-rotating twin-screw extruder at 100–120 °C with a screw speed of 60 rpm, ensuring homogeneous dispersion of the filler without thermal degradation. The extruded material was pelletized and then compression-molded using a hydraulic press with heated plates from Remi-Plast (Czerwonak, Poland) at 120 °C under 15 MPa for 5 min. The resulting sheets, with a thickness of 0.5 mm, were then cut into specimens for further characterization. This method ensured the controlled preparation of biodegradable polymer composites, allowing for reproducibility in material processing and subsequent property evaluation.

### 2.3. Methods

The composition of the copolyester was verified using ^1^H NMR spectroscopy (Bruker DPX 400 (Karlsruhe, Hamburg, Germany) spectrometer equipped with a 5 mm 1H/BB inversion head, operating at 400.13 MHz). A sample spin rate of 20 Hz was used. The sample was prepared by dissolving approximately 20 mg of polymer in 0.6 mL of deuterated chloroform (ARMAR Chemicals, Dottingen, Switzerland, 99.8% D atom). The internal standard was tetramethylsilane.

The water contact angle (WCA) was measured using the sessile drop method with deionized water on a DSA100 Drop Shape Analyzer (KRÜSS, Heidelberg, Germany). A 2 µL water droplet was placed on the sample surface, and the contact angle was recorded immediately. Measurements were repeated five times per sample, and the average value was reported.

Tensile tests were carried out on an Instron 3366 universal testing machine at a crosshead speed of 100 mm/min, following the PN-EN ISO 527-1:1998 standard. The samples were prepared using a pneumatic press and measured prior to testing. The results were averaged from 7 specimens.

The surface morphology of the samples before and after biodegradation was examined using a Leica S9I (Kawaska, Zalesie Gorne, Poland) stereoscopic microscope. Additionally, the topography and roughness of the degraded samples were analyzed with a VK-9710 3D laser scanning microscope (Keyence, Osaka, Japan) at magnifications of 5×, 20×, and 50×.

Fourier-transform infrared spectroscopy was performed using a Bruker Alpha spectrometer (Karlsruhe, Germany) in ATR mode (ATR FT-IR). Spectra were recorded in the range of 4000–400 cm^−1^ to assess the chemical structure and possible degradation-induced changes.

The thermal transitions of the obtained materials were evaluated using a TA Instruments DSC Q2500 Discovery differential scanning calorimeter (DSC) (TA Instruments Inc., New Castle, DE, USA). The measurements were performed in a nitrogen atmosphere over the temperature range of −90 to 150 °C, with heating and cooling rates set at 10 °C/min. The glass transition temperature (T_g_) was determined as the midpoint of the transition during the second heating step to eliminate thermal history effects.

DMTA measurements were performed using a DMA Q800 apparatus from TA Instruments (TA Instruments Inc., New Castle, DE, USA) in the temperature range of −100 to 150 °C under liquid nitrogen. The analysis provided the storage modulus (E′), loss modulus (E″), and tan δ.

Thermal gravimetric analysis (TGA) was carried out using the TGA DTA-Q600 SDT (TA Instruments Inc., New Castle, DE, USA) model in argon with a heating rate of 10 °C/min. 

### 2.4. Biodegradability Test—Soil Burial Method

The soil burial test was conducted to evaluate the biodegradability of the polymer composites under mesophilic conditions (30 °C) in accordance with the EN 14806:2005 standard. The test was performed using a microbiological incubator to maintain controlled environmental conditions.

**Figure 2:**
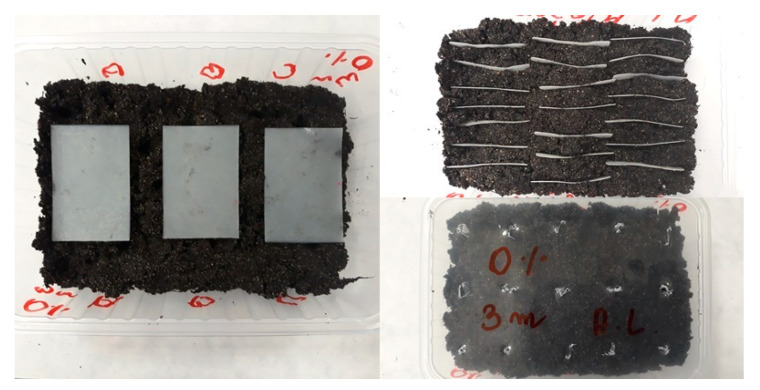
Preparation of samples for soil degradation testing.

Prior to testing, rectangular (6 × 4 cm) and dumbbell-shaped specimens (Figure 2) were prepared from both the composite films and unmodified PBS-DLA copolyester. All samples were disinfected using 70% isopropyl alcohol and precisely weighed. The incubation substrate consisted of a mixture of gardening soil (3 L), sand (1 L), distilled water (500 mL), and liquid fertilizer (10 mL). Sand was added to improve the soil permeability and ensure the better access of microorganisms to the samples, while the fertilizer provided essential nutrients to support biological activity during biodegradation. Disinfected polypropylene containers were filled with a 3 cm thick layer of the substrate, on which the samples were placed. They were then covered with an additional 2 cm layer, ensuring a total substrate volume of 1 L. The containers were sealed with ventilated lids to allow gas exchange and incubated for 1, 2, and 3 months in an incubator at a controlled temperature of 30 °C to ensure stable biodegradation conditions.

During the test, the moisture level was maintained at 40% by regularly adding distilled water, as verified by periodic weighing of the containers with soil and samples. After the designated incubation period, the samples were removed, washed with distilled water to remove soil residues, disinfected with isopropyl alcohol, and weighed. They were then dried to a constant mass and reweighed to determine the mass loss (*D*), calculated using the following Equation (1):

(1) $$D=\frac{m_{0}-m_{d}}{m_{0}}\times 100\%$$

where *D*—weight loss [%], *m*₀—initial sample weight before the soil test, and *m_d_*—sample weight after incubation.

The degree of biodegradation was assessed based on the average percentage mass loss of the tested films. Photographic documentation of the samples was recorded at each stage of the study to observe visual changes in surface morphology.

## 3. Results and Discussion

### 3.1. Biodegradation Under Soil Conditions

**Figure 3:**
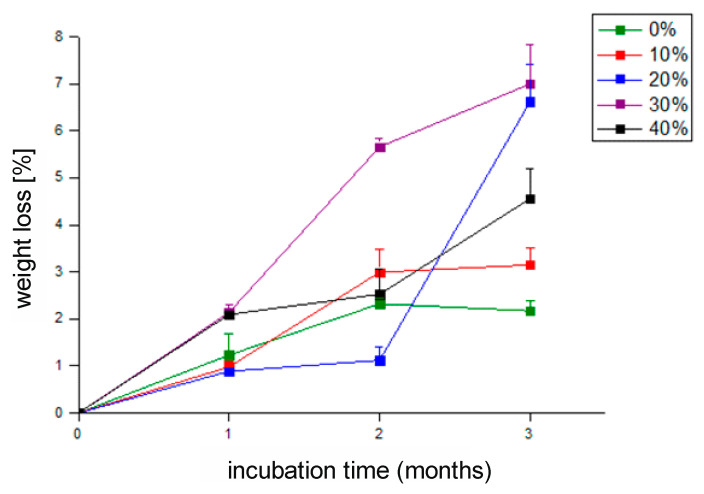
Weight loss of PBS-DLA composites with wood flour (0–40%) after soil testing.

Overall, PBS-DLA copolymers containing hydrophobic fatty acid experienced relatively slow degradation. For the unfilled PBS-DLA copolyester, the degradation rate was moderate during the first two months (Figure 3), whereas after three months, the mass loss reached only 2% and it was the lowest among all of the tested samples. Recent studies have confirmed that PBS degradation under mesophilic conditions (30 °C) is driven by microbial enzymatic activity, primarily through the action of esterases and lipases, which cleave ester bonds and facilitate polymer breakdown [8]. This aligns with our observations, suggesting that microbial activity contributed to the degradation process in soil conditions.

The incorporation of WF into the polymer matrix showed a marked effect on mass loss. After two months, the 20 wt.% WF composite exhibited the slowest biodegradation; however, after this period, the degradation process accelerated nearly sixfold. After three months of composting, the greatest mass loss (7%) was observed in the composite containing 30 wt.% wood flour (WF). During this period, the mass loss increased more than threefold, distinguishing this material from the other tested samples. The incorporation of 40 wt.% of WF did not further increase the mass loss.

Previous studies have shown that the biodegradation rate of wood–polymer composites (WPCs) generally increases with a higher content of cellulosic fillers, as they promote moisture absorption and microbial colonization [33]. However, as demonstrated in studies on WPC subjected to brown-rot fungi, the degradation of these materials does not always follow a linear pattern—in some cases, composites with a moderate lignocellulosic filler content degraded faster than those with a higher concentration [33]. A similar nonlinear relationship was observed in our PBS-DLA composites with wood flour, where the material containing 30 wt.% filler exhibited the highest mass loss, surpassing the value recorded for the 40 wt.% sample. Furthermore, a sudden acceleration of degradation was noted in the composite containing 20 wt.% filler after two months.

The lack of a clear trend may also be attributed to the distribution of the filler within the polymer matrix. It is possible that in the 30 wt.% WF composite, larger filler particle aggregates formed, which could have influenced the biodegradation behavior. Studies indicate that both the content and particle size of wood fillers significantly impact the mechanical properties of polymer composites, with smaller filler particles often leading to improved mechanical strength and better interfacial adhesion [34]. These factors could, in turn, influence the degradation process. In the case of a composite with 20% wt. WF, it was hypothesized that the wood flour particles were coated with a thin polymer film, limiting the access of enzymes in the early stages of the test, and then boosting the weight loss once the polymer was degraded and the cellulose particles were exposed to attack by soil enzymes.

Nevertheless, the initial assumption that the addition of wood flour accelerates the decomposition process has been confirmed, emphasizing the beneficial effect of this filler on the biodegradation of the tested materials. These results confirm that the biodegradation process is strongly influenced not only by filler content but also by particle size and filler dispersion within the matrix, as observed previously in similar lignocellulosic polymer composites [28,33,34].

At the end of the soil test, after calculating mass loss, the fungal growth on the sample surfaces was assessed visually using a six-point scale (0–5), where 0 indicates no growth and 5 represents intense fungal colony development:

0 points—no growth on the sample surface.

1 point—no growth on the surface, slight growth on some edges.

2 points—no growth on the surface, slight growth on all edges.

3 points—no growth on the surface, intense growth on all edges.

4 points—slight growth on the surface, intense growth on edges.

5 points—intense growth on both the surface and edges of the sample.

The fungal growth assessment depending on the wood flour content and the incubation time was as follows:

0 wt.% WF—gradual increase from 0 points (first month) to 4 points (third month).

10 wt.% WF—slight growth after one month (1 point), then intensification (4 points in the second month, 5 points in the third month).

20 wt.% WF—rapid colony growth: 2 points (first month), 4 points (second month), 5 points (third month).

30 wt.% WF—significant growth after one month (4 points), reaching the maximum level (5 points in the second and third month, respectively).

40 wt.% WF—similar to the 30 wt.% WF composite (4 points in the first month, 5 points from the second month onward).

### 3.2. Surface Assessment—Microscopic Analysis

**Figure 4:**
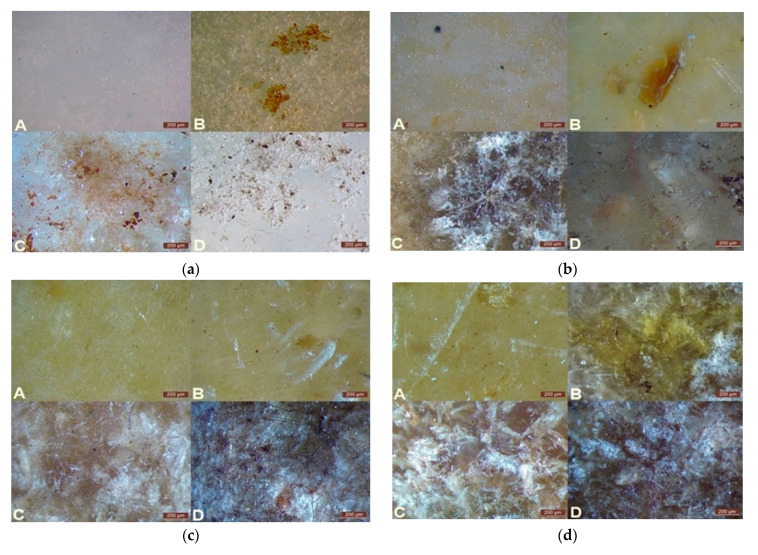

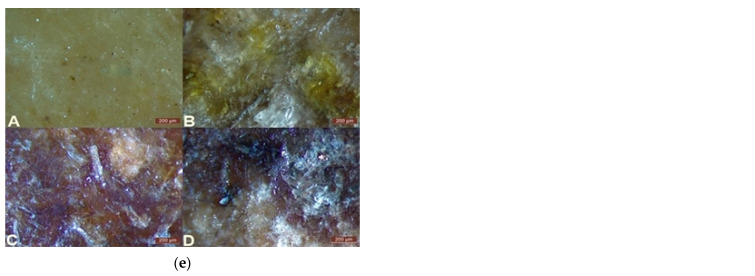
Surface analysis of PBS-DLA composites with different wood flour contents: A—before degradation, B—after one month, C—after two months, D—after three months of degradation for 0% (**a**), 10% (**b**), 20% (**c**), 30% (**d**), and 40% (**e**) wood flour content. Scale bars—200 μm.

Stereoscopic microscopy was used to analyze surface changes in the tested materials before and after degradation. The obtained images allowed for the evaluation of the structural integrity of WPCs and the identification of surface defects such as pits, cracks, and discolorations. Particular attention was given to the dispersion of the filler in the polymer matrix and the progressive changes occurring on the surface during biodegradation. These results were presented in Figure 4, where the surface of PBS-DLA composites with different wood flour contents (0%, 10%, 20%, 30%, and 40 wt.%) was analyzed before degradation and after one, two, and three months of degradation.

Before degradation (Figure 4a), microscopic images revealed the non-uniform distribution of wood flour (WF) in the polymer matrix (B, C, D). In many cases, large aggregates of filler particles were visible, suggesting incomplete mixing during processing and indicating potential technological challenges in sample preparation. Ideally, well-dispersed filler should not be detectable under an optical microscope.

As biodegradation progressed, the surface topography significantly changed. In the early stages, material cohesion began to deteriorate, leading to the formation of small pits and microcracks. Over time, these defects expanded, resulting in the progressive disintegration of the samples. The most pronounced changes were observed in composites containing 30 wt.% and 40 wt.% WF, which exhibited numerous deep cracks (Figure 4d,e). In some cases, material fragmentation was visible, indicating the high susceptibility of these composites to biodegradation. In contrast, the unfilled PBS-DLA copolyester (Figure 4a, A) and 10 wt.% (Figure 4b, A) WPC showed only minor surface changes, limited to small holes and a slight increase in the roughness.

One of the most noticeable effects of degradation was the color change of the samples. Significant darkening of the surface was observed, especially in samples with a high WF content, which can be attributed to intense microbial activity. The appearance of greenish-brown stains suggests the formation of bacterial and fungal biofilms, which may further accelerate biodegradation through enzymatic polymer breakdown. This phenomenon has been previously described in the context of fungal colonization of lignocellulosic materials, where fungi not only degrade the polymer structure but also alter the optical properties of the surface [33,35]. In particular, brown-rot fungi have been reported to cause oxidative modifications leading to discoloration before structural degradation occurs [35]. A similar effect was observed in PBS-DLA composites, particularly those containing 30 wt.% and 40 wt.% WF, while in samples with lower WF content, the color changes were less intense.

The observed surface changes strongly correlated with mass loss results, confirming that degradation was the most intense in composites with a higher WF content. Filler aggregates acted as localized structural weak points, contributing to uneven material breakdown. Additionally, the moisture-retaining properties of WF may have increased the susceptibility of these composites to microbial attack, explaining the more pronounced degradation effects in samples with higher filler concentrations.

The microscopic analysis results align with the mass loss data (Figure 3), which demonstrated that composites with a higher WF content exhibited a faster degradation rate. The samples that experienced the most significant mass loss after three months of testing also showed the most advanced surface deterioration, indicating a strong relationship between chemical degradation and structural disintegration.

Stereoscopic microscopy provided key evidence confirming the progressive nature of the biodegradation process, offering valuable insight into how the composition of the material influences its susceptibility to breakdown in soil conditions. The presented observations clearly indicate that while WF accelerates biodegradation, it also leads to heterogeneous material degradation, the intensity of which is closely related to the dispersion level of the filler within the polymer matrix.

### 3.3. Surface Topography—Laser Scanning Microscopy

To assess surface topography changes resulting from the biodegradation process, laser confocal microscopy was used. This technique enabled the analysis of the surface roughness of the tested samples both before and after exposure to soil conditions. Particular attention was given to changes in surface parameters and their correlation with the degree of material degradation.

**Figure 5:**
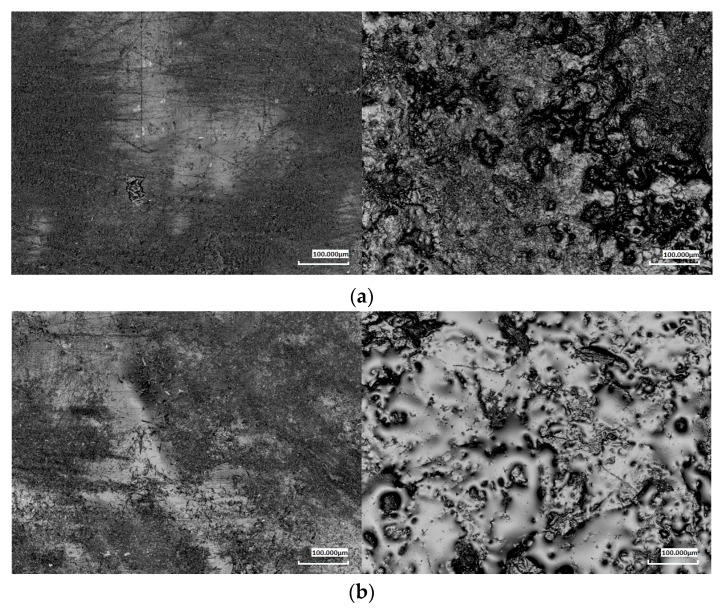

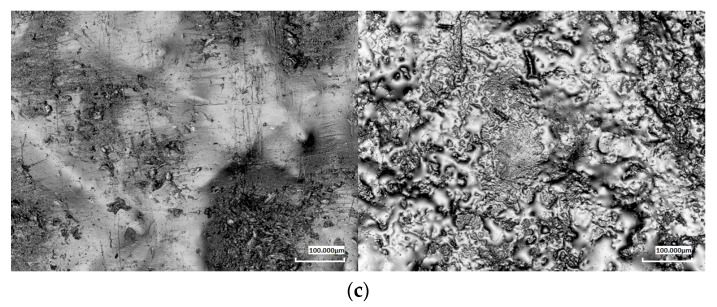
Changes in the topography of the samples due to biodegradation (on the left—before degradation; on the right—after 3 months of degradation) for PBS-DLA with 0% (**a**), 20% (**b**), and 40% (**c**) wood flour. Magnification: 5×.

Microscopic analysis performed at 5×, 20×, and 50× magnifications allowed for a detailed evaluation of the topographical changes during biodegradation for two representative composites: 20% and 40 wt.% (Figure 5). The results showed significant differences between the samples before and after three months of exposure to the soil environment. The neat PBS-DLA samples exhibited a relatively smooth surface, while materials with wood flour (WF) exhibited a noticeable initial roughness due to the presence of the filler. In particular, composites with a high WF content (40%) displayed surface irregularities related to the non-uniform distribution of the filler within the polymer matrix.

After three months of biodegradation, a significant increase was observed in the values of the roughness parameters—the arithmetic mean (Ra) and root mean square (Rq), which describe the Ra and Rq deviation of the surface profile from the mean line, respectively. The highest roughness increase was found for PBS-DLA and PBS-DLA + 20% WF samples, while the changes in PBS-DLA + 40% WF were less pronounced, indicating the formation of a polymer skin on the surface covering protruding agglomerates. For example, at 5× magnification, the Ra values increased from 16.43 µm to 60.77 µm in PBS-DLA, from 24.93 µm to 63.46 µm in PBS-DLA + 20% WF, and from 24.68 µm to 40.47 µm in PBS-DLA + 40% WF, respectively.

Microscopic images confirmed that the surface of the samples became significantly more irregular after biodegradation. In PBS-DLA and PBS-DLA + 20% WF, numerous pits, indentations, and grooves were observed, indicating intense material degradation. In PBS-DLA + 40% WF, changes were less apparent, which may result from the polymer skin formation on the surface responsible for the restricted access of enzymes and fungi to the cellulose filler.

The obtained results align with the mass loss data. The samples that exhibited the highest mass loss also showed the greatest increase in roughness. The increase in surface irregularities likely promoted greater water adsorption and microbial adhesion, further accelerating biodegradation. Notably, our results clearly demonstrate that in PBS-DLA + 20% WF, a significant roughness increase was observed, coinciding with a sudden acceleration of degradation after two months. This suggests that microstructural changes at the surface level play a key role in determining the degradation rate of these composites.

Conversely, PBS-DLA + 40% WF exhibited a smaller increase in roughness, suggesting that at a higher filler content, the wood particles tend to form agglomerates, while the polymer matrix partially migrates to the surface. This effect may reduce the direct exposure of the filler to microbial activity, potentially slowing down the surface degradation process.

The surface topography analysis provided essential insights into the impact of biodegradation on the structure of the tested materials. The results confirm that increased surface roughness is both a consequence and a potential factor accelerating biodegradation, emphasizing the critical role of surface morphology in polymer degradation processes. The observed relationship between roughness increase and mass loss highlights the importance of the composite microstructure in determining material durability under natural conditions.

### 3.4. Wettability and Hygroscopic Behavior

Due to the irregularity of the sample’s surface after degradation, it was not possible to perform contact angle measurements. After three months of biodegradation, the surface of the samples did not allow for proper drop placement, making it impossible to obtain reliable results.

**Figure 6:**
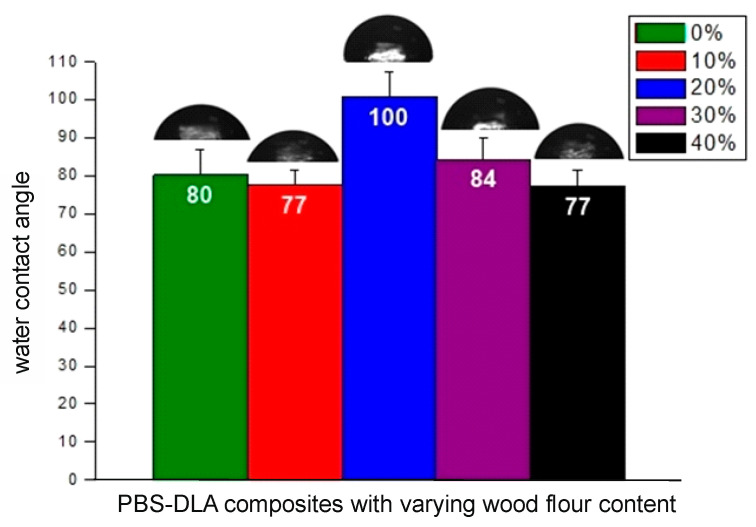
Average contact angle values of the composites before degradation. Percentages in legend are referring to the amount of WF.

Therefore, the wettability of the PBS-DLA copolyester and its composites was evaluated based on contact angle measurements conducted before degradation (Figure 6). The results confirmed that the neat PBS-DLA copolyester exhibited moderate hydrophilicity, with an initial contact angle of 80°. The incorporation of wood flour (WF) influenced the surface properties, leading to a general trend of decreasing contact angle values, indicating improved wettability. However, an unexpected increase in contact angle was observed for the 20% WF composite, which exhibited an average value of 100°, classifying the material as hydrophobic. However, given the highest surface roughness observed for this WPC, this behavior can be explained according to the Cassie–Baxter law indicating incomplete wetting of the surface by water [36]. On the other hand, the formation of a skin effect may have restricted the direct contact of the WF with water molecules, reducing the material’s ability to effectively absorb moisture (see Figure 5b).

A similar behavior of an increase in contact angle—however, not so pronounced—was observed for 30 wt.% WF (compared to 10 wt.% WF and 40 wt.% WF composites). It may also be attributed to the Cassie–Baxter effect, where air is trapped in microcavities formed between filler aggregates, reducing the effective contact area between the water droplet and the composite surface. Such behavior is well recognized in biomimetic material research, notably in studies on hydrophobic surfaces inspired by nature, such as the lotus leaf surface [36,37]. Additionally, during composite processing, polymer matrix migration toward the surface could result in the formation of a thin PBS-DLA-rich skin layer, further limiting the direct contact of hydrophilic WF particles with water molecules. Similar mechanisms have been described in cellulose-based nanocomposites [38].

The reduction in contact angle in the 10% and 40% WF composites suggests that the cellulose-based filler improved the hydrophilicity of the material. This was expected, as wood flour contains cellulose hydroxyl groups capable of forming hydrogen bonds, increasing its affinity to water.

Since biodegradation is closely linked to water absorption, the limited wettability of the 20% WF composite correlates with its slower initial biodegradation rate (up to 2 months). Hydrophobic surfaces hinder the transport of the hydrolytic enzymes responsible for polymer breakdown, explaining why this composite degraded at a slower rate during the first two months. However, once the material structure began to break down, water absorption likely increased, leading to a sudden acceleration of the degradation process. In contrast, composites with higher hydrophilicity degraded more rapidly, supporting the hypothesis that greater water absorption promotes microbial activity.

### 3.5. Functional Group Analysis—FTIR

The chemical structure of the synthesized PBS-DLA copolyester was analyzed with ^1^H NMR spectroscopy, and very good agreement was obtained between the theoretical (50 wt.%) and experimental (48.7 wt.%) content of PBS hard segments (see the Supporting Materials, Figure S1).

**Figure 7:**
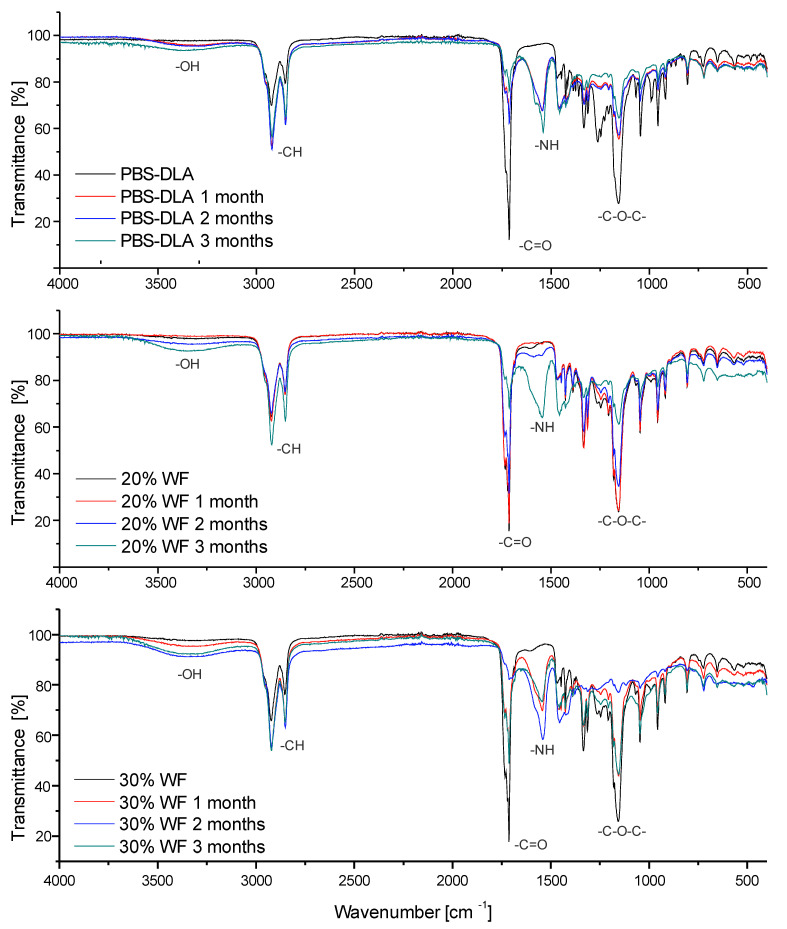
FTIR spectra of PBS-DLA composites with 0%, 20%, and 30% wood flour content after different incubation times (0, 1, 2, and 3 months).

The chemical structure of the PBS-DLA copolyester and its composites was mainly analyzed using Fourier-transform infrared spectroscopy. The objective was to identify characteristic functional groups and track structural changes occurring in the material during biodegradation of the neat material and composites up to 30 wt.% of WF (Figure 7).

The spectra of neat PBS-DLA exhibited characteristic absorption bands corresponding to its ester structure. The most significant bands appeared at 2922–2850 cm^−1^ (C–H stretching vibrations), 1713 cm^−1^ (C=O stretching from esters), and 1155 cm^−1^ (C–O–C vibrations), confirming the presence of the aliphatic polyester backbone.

After three months of biodegradation, noticeable changes were observed in the spectra of both neat PBS-DLA and its composites with wood flour (WF). A decrease in the C=O stretching peak at 1713 cm^−1^ suggested the hydrolytic breakdown of ester bonds. Simultaneously, an increase in the intensity of hydroxyl (–OH) bands around 3391 cm^−1^ was recorded, indicating the accumulation of degradation by-products. Additionally, in some samples, new absorption bands appeared around 1540–1555 cm^−1^ (N–H bending vibrations), despite the absence of nitrogen in the original PBS-DLA structure.

The unexpected appearance of nitrogen-containing functional groups after biodegradation suggests microbial involvement in the decomposition process. Although PBS-DLA does not contain nitrogen in its original structure, microbial activity can introduce new functional groups through enzymatic action, biofilm formation, or the adsorption of organic compounds from the environment, as commonly observed in biodegradation processes [39]. One possible explanation is enzymatic degradation, where microbial enzymes produced during polymer breakdown contributed to the formation of these signals. Another possibility is the adsorption of nitrogen-containing organic compounds from the soil environment, including microbial metabolites or amino acids, which could adhere to the material’s surface. It is also likely that fungi developing on the samples played a role. The detection of amide-related signals in the degraded samples may indicate the presence of chitin and chitosan, structural components of fungal cell walls. These substances, known for their nitrogen-containing functional groups, could have contributed to the spectral changes observed after biodegradation. The FTIR spectra of composites with 20% and 30% WF exhibited the most significant changes. In the 20% WF composite, the appearance of nitrogen-related bands was delayed until the third month of degradation, which aligns with its initially slower biodegradation rate. In contrast, the 30% WF composite showed an earlier and more intense increase in N–H bands, consistent with its higher degradation rate compared to other materials.

The FTIR results strongly correlate with other findings related to mass loss, wettability, and surface roughness, confirming that PBS-DLA degradation is influenced not only by its chemical composition but also by microbial activity in the surrounding environment.

### 3.6. Mechanical Properties—Tensile Strength

**Figure 8:**
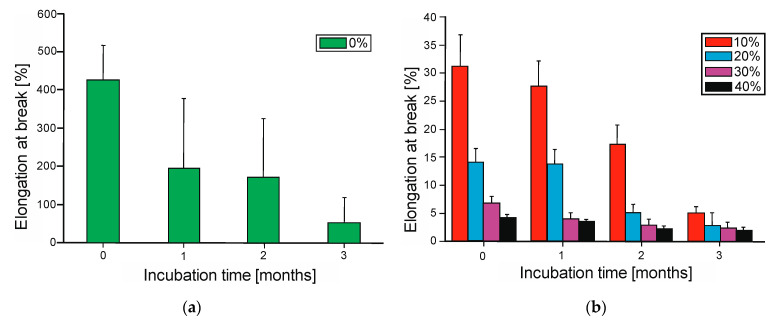
Elongation at break for (**a**) PBS-DLA (0% wood flour) and (**b**) PBS-DLA composites with different wood flour contents (10%, 20%, 30%, 40%) after different incubation times (0, 1, 2, and 3 months).

**Figure 9:**
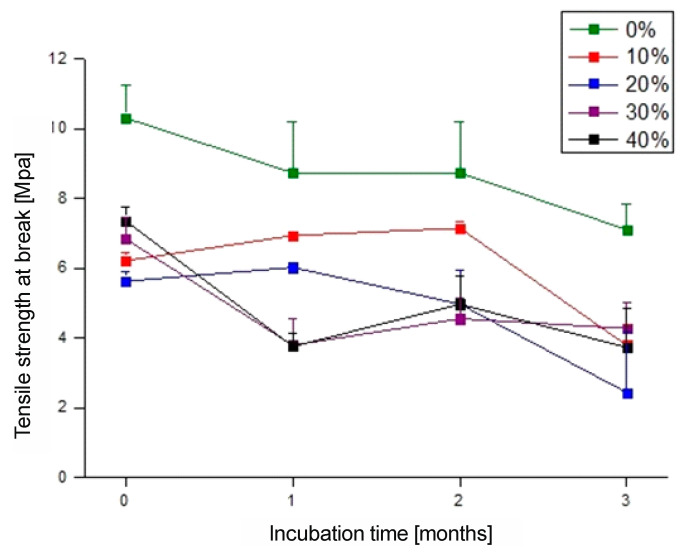
Tensile strength at break of PBS-DLA and PBS-DLA/WF composites as a function of incubation time in soil.

The analysis of mechanical properties revealed significant differences in the behavior of the materials before and after biodegradation (Figure 8 and Figure 9). The neat PBS-DLA copolyester exhibited high elasticity (Figure 8a) which progressively dropped after three months of degradation. The addition of wood flour (WF) significantly influenced the mechanical properties, leading to a decrease in elongation at break (Figure 8b) and changes in tensile strength (Figure 9). After three months of biodegradation, all samples showed a substantial decline in mechanical performance, which aligned with previous observations regarding mass loss, surface roughness, and wettability.

As can be seen from Figure 8a and Figure 9, respectively, the elongation at break for the neat PBS-DLA copolymer reached 426%, indicating good elasticity, and the tensile strength was 10.3 MPa before biodegradation. The introduction of WF caused a significant reduction in these parameters. Specifically, the elongation at break decreased nearly fourteenfold to 31.2% (Figure 8b) when 10% WF was introduced, and the tensile strength dropped to 6.21 MPa. The 20% WF composite exhibited a further reduction in elongation at break to 14%, while tensile strength dropped to 5.62 MPa. Samples containing 30% WF showed a drastic reduction in elongation to 6.8% and a slight increase in tensile strength (6.83 MPa). A clear reinforcing effect was observed for the 40% WF composite, where the tensile strength increased to 7.35 MPa but the material exhibited extreme brittleness, with elongation dropping to 4.2%.

After three months of biodegradation, all samples experienced a significant loss in mechanical properties. The elongation at break of neat PBS-DLA (Figure 8b) dropped by nearly 90%, reaching only 53.9%, while the tensile strength decreased to 7.10 MPa (Figure 9). The most drastic changes were observed in the WF composites. The 10% WF composite exhibited a tensile strength of 3.78 MPa, with elongation decreasing to 4.93%, indicating progressive degradation of the polymer matrix. The 20% WF composite showed even lower values, with the tensile strength dropping to 2.43 MPa and elongation to only 2.84%. Interestingly, the 30% WF composite had a higher tensile strength (4.27 MPa) than the 20% WF sample, though its elongation was only 2.40%, suggesting high stiffness and brittleness after degradation. Despite initially having the highest tensile strength, the 40% WF composite degraded to 3.73 MPa, with minimal elongation of 2.04%.

The obtained mechanical results strongly correlated with the described biodegradation analyses. The most significant loss of mechanical properties occurred in the 20% WF composite, which aligns with its sudden acceleration of biodegradation after two months. Its lower water absorption, confirmed by the higher contact angle, may have initially limited enzyme access, but once the structure weakened, degradation proceeded rapidly. In contrast, the 30% WF composite exhibited greater mechanical stability, which was consistent with the previous findings on surface roughness and wettability. Meanwhile, the PBS-DLA + 40% WF composite, despite initially having a greater tensile strength, became extremely brittle after degradation, likely due to the excessive filler content disrupting the polymer matrix continuity and reducing interfacial adhesion, and ultimately to the deterioration of mechanical properties.

### 3.7. Thermal and Thermomechanical Analysis

**Figure 10:**
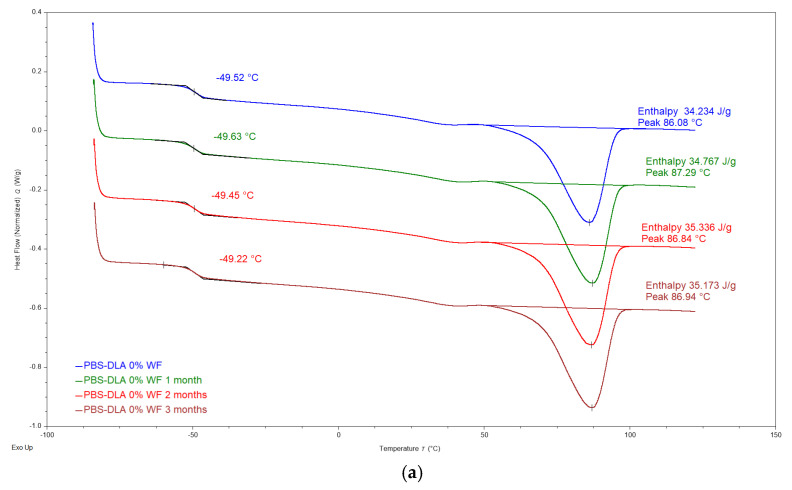

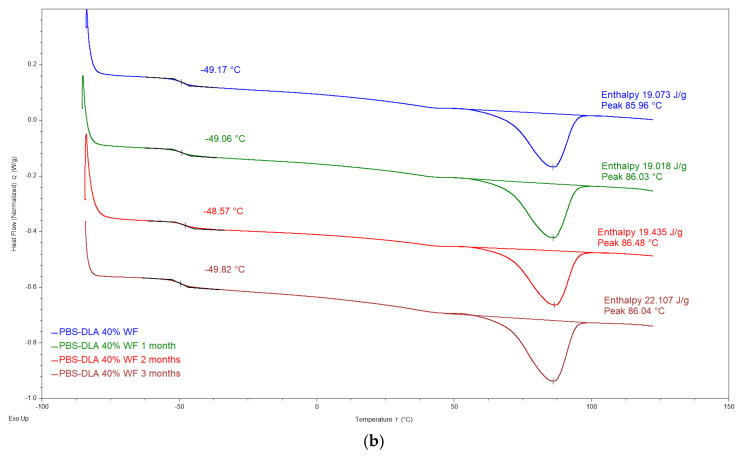
DSC thermograms of the second heating cycle for (**a**) neat PBS-DLA copolymer and (**b**) PBS-DLA + 40% WF composite, before and after biodegradation.

The DSC analysis did not reveal significant changes in the thermal properties of PBS-DLA and its wood flour (WF) composites after biodegradation (Figure 10). The glass transition temperature (T_g_) oscillates around −49.4 °C, regardless of the WF content and degradation time. Δc_p_ values show a slight decrease, but since enthalpy is calculated relative to the total sample mass, including the WF phase that does not undergo the glass transition, the observed changes in Δc_p_ cannot be directly interpreted as restricted chain mobility.

The melting temperature (T_m_) and crystallization temperature (T_c_) remain largely unchanged, suggesting that the crystalline phase of the polymer is not significantly hindered during biodegradation. Representative DSC thermograms, as presented in Figure 10, illustrate the behavior of PBS-DLA and the 40% WF composite before and after degradation. The lack of pronounced changes in DSC characteristics confirms that degradation is primarily a surface process and does not lead to polymer crystalline structure breakdown on a macromolecular level, which is consistent with previous morphological observations.

**Figure 11:**
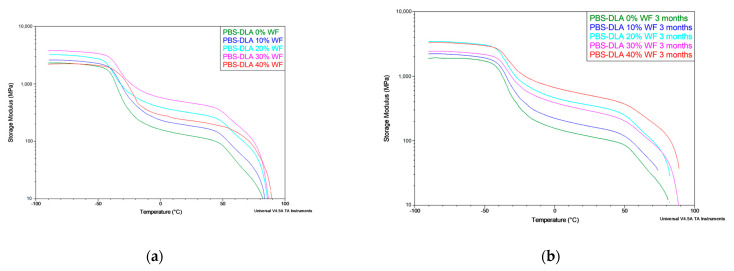
Storage modulus (E′) as a function of temperature for PBS-DLA and PBS-DLA composites with different wood flour (WF) contents before biodegradation (**a**) and after 3 months of biodegradation (**b**).

DMTA results further confirmed the stability of the crystalline phase of the polymer (hard segments), revealing no significant differences in the storage modulus (E′) and loss modulus (E″) after biodegradation (Figure 11). Despite the deterioration in mechanical properties observed in tensile tests, DMTA did not indicate the structural degradation of a polymer matrix, which supports the conclusion that biodegradation primarily affects the material’s surface. The effect of WF content on the storage modulus (E′) is illustrated in Figure 11a, confirming the reinforcing effect up to 30% WF and its subsequent decrease at 40% WF. Furthermore, the comparison before and after biodegradation (Figure 11b) indicates that degradation has minimal impact on E′, reinforcing the hypothesis of surface erosion rather than bulk material breakdown.

The combined results of DSC, DMTA, and mechanical tests confirm that PBS-DLA is a thermoplastic elastomer. The material exhibits high elasticity, as evidenced by its stable thermal transitions and mechanical response even after the biodegradation test for 3 months. These findings further confirm that PBS-DLA is a segmented thermoplastic elastomer (TPE), where the soft amorphous phase is formed by dilinoleic acid (DLA) ester segments, while the hard phase consists of crystallizable PBS domains. This unique phase structure provides both elasticity and thermoplastic processability, ensuring the material retains its mechanical functionality after prolonged degradation. The addition of WF enhances the mechanical properties of the composite up to a 30% content, after which the reinforcing effect diminishes. At 40% WF, a decrease in the storage modulus (E′) and overall mechanical performance is observed, indicating that excessive filler content may disrupt the polymer matrix continuity and reduce interfacial adhesion.

Furthermore, the degradation behavior of PBS-DLA and its composites strongly supports a surface erosion mechanism rather than bulk degradation. The stability of T_g_, T_m_, and thermomechanical properties over time, along with the significant loss of mechanical performance in tensile tests, indicates that biodegradation predominantly affects the outer layers of the material. This observation is consistent with previous studies on the surface morphology of degraded samples, where an increase in surface roughness, the formation of microcracks, and microbial activity suggested that degradation progresses through surface erosion rather than bulk material breakdown. These findings reinforce the conclusion that the biodegradation of PBS-DLA and its composites predominantly occurs through surface erosion, leaving the bulk polymer structure largely intact.

**Figure 12:**
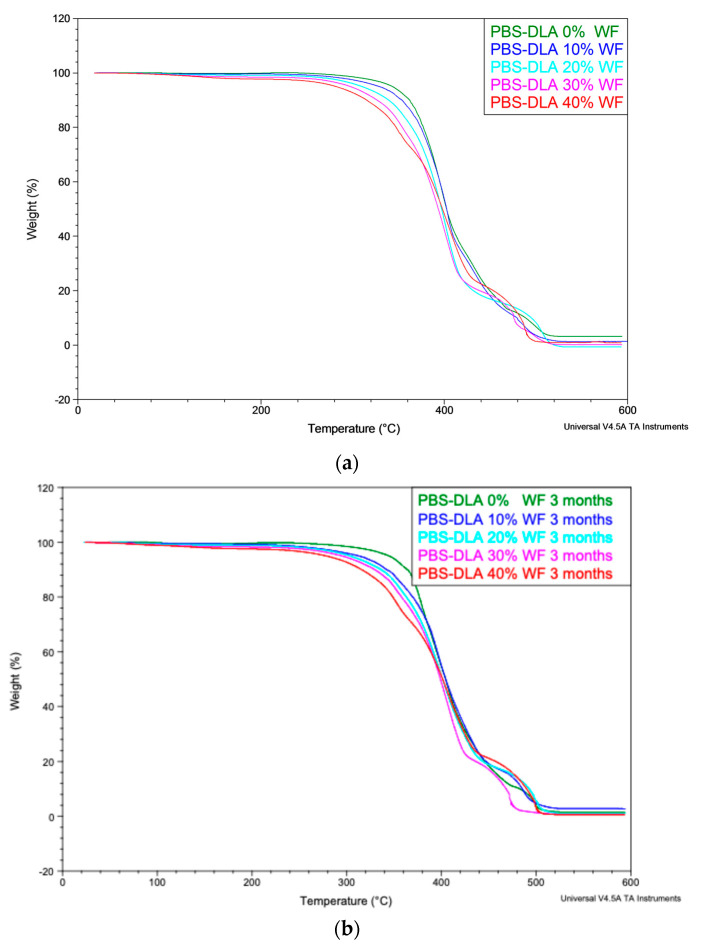
Thermogravimetric analysis (TGA) curves of PBS-DLA copolymer and PBS-DLA/WF composites containing 0–40 wt.% wood flour: (**a**) samples before biodegradation, (**b**) samples after three months of soil burial.

To further investigate the thermal behavior of the PBS-DLA copolymer and its composites after biodegradation, thermogravimetric analysis (TGA) was conducted before and after the soil burial test. Figure 12a presents the TGA curves for all composites (0–40 wt.% WF) before degradation, while Figure 12b shows the corresponding curves after three months of biodegradation. The initial degradation temperature of the neat PBS-DLA copolymer before and after degradation exceeded 350 °C, confirming thermal stability and no changes in molecular weight. The incorporation of wood flour slightly reduced the onset degradation temperature down to 315 °C due to the lower thermal stability of lignocellulosic filler.

Importantly, no significant differences in thermal degradation behavior were observed after biodegradation. The TGA profiles remained largely unchanged, indicating that biodegradation primarily affects the surface of the material and does not compromise the thermal stability or bulk structure of the PBS-DLA copolymer matrix or its molecular weight. These results, in combination with DSC, DMTA, and FTIR data, confirm that while surface degradation and filler decomposition reduce the mechanical performance, the copolymer matrix remains thermally and structurally stable at the macroscopic level over three months of degradation experiments.

## 4. Conclusions

This study demonstrated that the PBS-DLA copolymer is a segmented thermoplastic elastomer (TPE), and both the copolyester and its composites with wood flour (WF) are biodegradable. The effects of degradation in soil were primarily observed on the surface, while the bulk structure of the material remained largely intact.

Microscopic analyses (stereomicroscopy and laser profilometry) confirmed the surface character of the degradation. A roughness increase, microcrack formation, and enhanced porosity were particularly evident in composites with a higher WF content, indicating that cellulose-based fillers promote biodegradation by facilitating moisture absorption and microbial colonization.

ATR FTIR spectroscopy further confirmed the biodegradability of the materials, revealing changes in the chemical structure of polymer matrix. Oxidation and the hydrolytic cleavage of ester bonds were detected, with more pronounced changes in the presence of WF, supporting the hypothesis of progressive surface erosion.

Mechanical testing provided additional evidence of degradation, as all materials exhibited a reduction in tensile strength and elongation at break after three months. The most pronounced deterioration occurred in composites with 20% WF, where degradation appeared to accelerate after two months. However, composites with up to 30% WF retained a moderate mechanical performance, balancing reinforcement with controlled biodegradation.

DSC and DMTA analyses confirmed that the PBS-DLA copolymer retains its thermoplastic elastomeric behavior after three months of biodegradation. The storage modulus (E’) remained stable, while only minor variations in melting and crystallization temperatures were observed. Additionally, thermogravimetric analysis (TGA) showed no significant changes in the thermal degradation behavior of the PBS-DLA copolymer and its composites after biodegradation, indicating that the bulk polymer matrix does not undergo degradation or molecular weight reduction during soil exposure for 3 months.

These findings reinforce the hypothesis of surface erosion rather than a bulk degradation mechanism and confirm the structural stability of the polymer matrix.

The results confirm that the PBS-DLA copolymer and its composites are biodegradable segmented thermoplastic elastomers, where surface degradation occurs without significant structural modifications to the polymer matrix.

## Data Availability

The raw data supporting the conclusions of this article will be made available by the authors on request.

## Supplementary Materials

The following supporting information can be downloaded at https://www.mdpi.com/article/10.3390/polym17070883/s1, Figure S1: ^1^H NMR spectrum of PBS-DLA copolymer; Table S1: Thermal properties of PBS/DLA and PBS/DLA + WF composites determined by DSC.
