# Supplementary material for: Biodegradable Polymer Composites Based on Poly(butylene succinate) Copolyesters and Wood Flour
Source: Polymers (Basel). 2025 Mar 26;17(7):883. doi: 10.3390/polym17070883 (PMC11991324; doi:10.3390/polym17070883)
Supplement: Supplementary file 1 [file polymers-17-00883-s001.zip › polymers-3519133-supplementary.pdf]

Biodegradable Polymer Composites Based on Poly(butylene succinate) Copolyesters and Wood Flour

Agnieszka Kozłowska, Krzysztof Gorący, Miroslawa El Fray *

Department of Polymer and Biomaterials Science, Faculty of Chemical Technology and Engineering, West Pomeranian University of Technology, Al. Piastow 45, 71-311 Szczecin, Poland; agnieszka.kozlowska@zut.edu.pl; kgoracy@zut.edu.pl; mirfray@zut.edu.pl
*Correspondence: mirfray@zut.edu.pl

Supporting Information

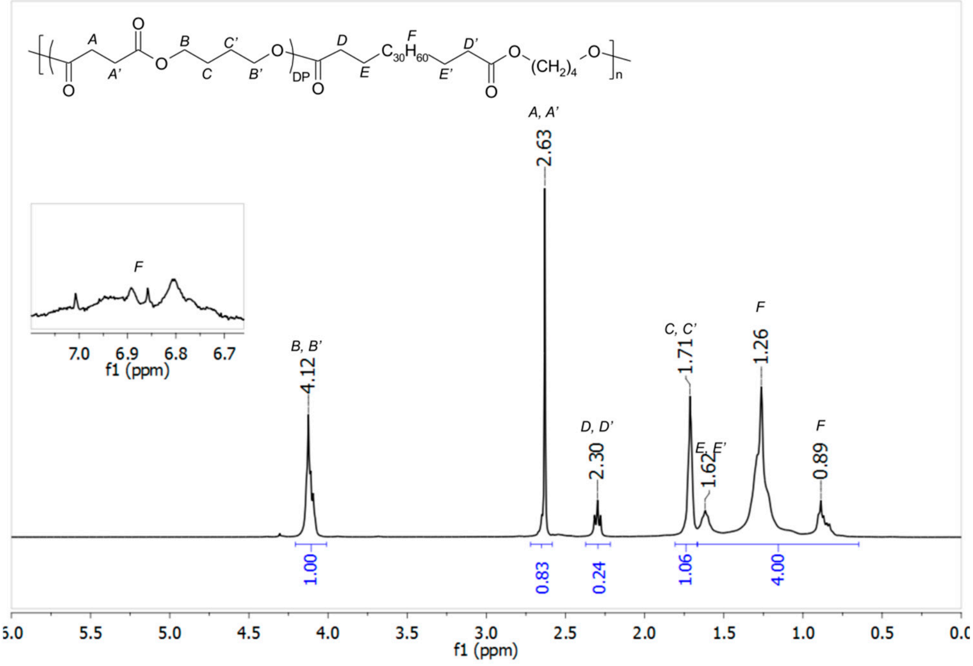

Figure S1. ^{1}H NMR spectrum of PBS-DLA copolymer

Weak signals in the range of 6.8–7 ppm originate from the aromatic systems of dilinoleic acid (DLA). The signals at 0.8–0.9 ppm are attributed to the terminal methyl protons of the branched structures within the soft segment derived from DLA, while the broad signal between 1.1 and 1.3 ppm corresponds to the methylene protons of the long aliphatic chains in the soft segment. Based on the integration of the signals at 2.30 ppm and 2.63 ppm, the content of hard segments (%H) in the copolymer was calculated using equation (1):

$$\%H = \frac{I_{2,63} \cdot M_H}{I_{2,63} \cdot M_H + I_{2,30} \cdot M_S} \cdot 100\% \quad (1)$$

where $I_{2.63}$ and $I_{2.30}$ represent the integrations at the respective chemical shifts, while M_H and M_S denote the molar masses of the hard and soft segments, respectively. The molar masses of the segments are as follows:

M_H = 172.18 g/mol, M_S = 626.59 g/mol.

The molar mass of the soft segments was calculated based on the molar mass of Pripol 1009 (dilinoleic acid), which is 572.50 g/mol. This value was determined using the acid number provided by the manufacturer (196 mg KOH/g), assuming the difunctionality of Pripol 1009.

The calculated content of the hard segments is %H = **48.7%**.

Table S1. Thermal properties of PBS/DLA and PBS/DLA + WF composites determined by DSC.

**material**	**T_g [°C]**	**Δc_p [J/(g·°C)]**	**T_m [°C]**	**ΔH_m [J/g]**	**T_c [°C]**	**ΔH_c [J/g]**
PBS/DLA before	-49,52	0,275	86,08	34,234	35,74	39,206
PBS/DLA after 1 month	-49,63	0,244	87,29	34,767	38,81	40,337
PBS/DLA after 2 month	-49,45	0,236	86,84	35,336	38,77	40,374
PBS/DLA after 3 month	-49,22	0,223	86,94	35,174	37,62	39,497
+ 10% WF before	-49,84	0,253	85,47	30,117	37,22	35,013
+ 10% WF after 1 month	-48,38	0,217	85,91	29,677	37,07	33,787
+ 10% WF after 2 month	-49,88	0,251	86,53	31,811	40,82	36,213
+ 10% WF after 3 month	-49,66	0,236	86,87	31,416	41,22	35,345
+ 20% WF before	-49,86	0,231	85,35	26,838	39,30	31,911
+ 20% WF after 1 month	-49,73	0,217	85,48	27,135	38,52	31,940
+ 20% WF after 2 month	-49,51	0,213	85,15	27,184	39,63	32,943
+ 20% WF after 3 month	-49,62	0,211	85,31	27,477	39,81	33,141
+ 30% WF before	-49,41	0,202	85,25	23,848	39,99	28,896
+ 30% WF after 1 month	-49,19	0,182	85,72	22,272	41,50	25,703
+ 30% WF after 2 month	-48,97	0,181	86,23	23,545	41,44	27,391
+ 30% WF after 3 month	-49,27	0,192	86,18	24,217	40,76	27,892
+ 40% WF before	-49,17	0,166	85,96	19,079	40,95	23,397
+ 40% WF after 1 month	-49,06	0,144	86,03	19,018	40,78	21,853
+ 40% WF after 1 month	-48,57	0,121	86,48	19,435	40,34	22,690
+ 40% WF after 3 month	-49,82	0,177	86,04	22,107	42,01	25,434

Tg [°C] – glass transition temperature

Δcp [J/(g·°C)] – specific heat capacity change

Tm [°C] – melting temperature

ΔHm [J/g] – melting enthalpy

Tc [°C] – crystallization temperature

ΔHc [J/g] – crystallization enthalpy.
